# Effect of sedative premedication with oral midazolam on postanesthesia care unit delirium in older adults: a secondary analysis following an uncontrolled before-after design

**DOI:** 10.1186/s13741-022-00253-4

**Published:** 2022-05-19

**Authors:** Karin Stuff, Elena Kainz, Ursula Kahl, Hans Pinnschmidt, Stefanie Beck, Franziska von Breunig, Rainer Nitzschke, Sandra Funcke, Christian Zöllner, Marlene Fischer

**Affiliations:** 1 Department of Anaesthesiology, University Medical Center Hamburg-Eppendorf, Hamburg, Germany; 2 Institute of Medical Biometry and Epidemiology, University Medical Center Hamburg-Eppendorf, Hamburg, Germany; 3 Department of Intensive Care Medicine, University Medical Center Hamburg-Eppendorf, Hamburg, Germany

**Keywords:** Delirium, Benzodiazepines, Perioperative care, Anesthesia, Premedication

## Abstract

**Background:**

Sedative premedication with benzodiazepines has been linked with prolonged recovery and inadequate emergence during the immediate postoperative period. We aimed to analyze the association between postanesthesia care unit (PACU) delirium and sedative premedication with oral midazolam.

**Methods:**

We performed a secondary analysis of prospectively collected data before (midazolam cohort) and after (non-midazolam cohort) implementation of a restrictive strategy for oral premedication with midazolam. From March 2015 until July 2018, we included patients 60 years and older, who underwent elective radical prostatectomy for prostate cancer. Exclusion criteria were contraindications to premedication with midazolam, preoperative anxiety, and a history of neurological disorders. Patients, who were scheduled for postoperative admission to the intensive care unit, were excluded. Between 2015 and 2016, patients received 7.5 mg oral midazolam preoperatively (midazolam cohort). Patients included between 2017 and 2018 did not receive any sedative medication preoperatively (non-midazolam cohort). The primary endpoint was the incidence of PACU delirium.

**Results:**

PACU delirium rates were 49% in the midazolam cohort (*n* = 214) and 33% in the non-midazolam cohort (*n* = 218). This difference was not statistically significant on multivariable logistic regression analysis (OR 0.847 [95% CI 0.164; 4.367]; *P* = 0.842). Age (OR 1.102 [95% CI 1.050; 1.156]; *P* < 0.001), the cumulative dose of sufentanil (OR 1.014 [95% CI 1.005; 1.024]; *P* = 0.005), and propofol-sufentanil for anesthesia maintenance (OR 2.805 [95% CI 1.497; 5.256]; *P* = 0.001) were significantly associated with PACU delirium.

**Conclusion:**

Midazolam for sedative premedication was not significantly associated with PACU delirium. The reduction in the incidence of PACU delirium throughout the study period may be attributable to improvements in perioperative management other than a more restrictive preoperative benzodiazepine administration.

**Supplementary Information:**

The online version contains supplementary material available at 10.1186/s13741-022-00253-4.

## Background

Postoperative delirium (POD) is a common complication after surgery and anesthesia that particularly affects patients older than 60 years (Inouye et al. 2014; Vlisides and Avidan 2019). It is defined as an acute cerebral dysfunction characterized by disturbance of consciousness, change in cognition or perception with fluctuating symptoms (American Psychiatric Association 2013). Postoperative delirium has been linked with subsequent cognitive impairment, increased institutionalization at hospital discharge, increased morbidity, mortality, and higher health care cost (Gottesman et al. 2010; Leslie et al. 2008; Sprung et al. 2017; Witlox et al. 2010).

Postanesthesia care unit (PACU) delirium is one subtype of POD that occurs early after surgery in the PACU (Card et al. 2015; Hernandez et al. 2017). Evidence from longitudinal studies suggests that PACU delirium is associated with the subsequent development of POD during hospital stay (Neufeld et al. 2013a; Sharma et al. 2005; Stukenberg et al. 2016; Zhang et al. 2020)*.*

To reduce preoperative anxiety and sympathetic activation, sedative premedication with benzodiazepines has been routinely used in periprocedural management (Bucx et al. 2016; Kain et al. 1997). However, accumulating evidence suggests that premedication with benzodiazepines may be a potential trigger for delayed time to extubation, prolonged postoperative recovery, and agitated emergence without improving self-reported patient experience after surgery (Lepousé et al. 2006; Maurice-Szamburski et al. 2015; Radtke et al. 2010). Owing to their neurocognitive adverse effects the American Geriatrics Society included benzodiazepines in the *Beers list for potentially inappropriate medication use in older adults* (American Geriatrics Society 2012)*.* Importantly, the *European Society of Anaesthesiology* guidelines on POD published in 2017 recommend a restrictive use of sedative premedication with benzodiazepines (Aldecoa et al. 2017).

In accordance with the novel recommendations, the liberal use of premedication with benzodiazepines has been restricted at our institution in 2017. The aim of this before-after secondary analysis was to compare the incidence of PACU delirium between a liberal and a restrictive benzodiazepine strategy in patients scheduled for radical prostatectomy. We hypothesized that premedication with midazolam would be associated with a higher incidence of PACU delirium.

## Methods

### Study design

We performed a secondary analysis following an uncontrolled before-after design to compare the incidence of PACU delirium between patients who received midazolam preoperatively (study 1: midazolam cohort) and patients who did not receive any sedative medication preoperatively (study 2: non-midazolam cohort). The midazolam cohort represents a convenience sample of 347 patients who had been enrolled in a prospective observational study (study 1) between January 2015 and March 2016 to compare the incidence of PACU delirium between open retropubic and robot-assisted radical prostatectomy (Beck et al. 2020b). Between November 2017 and October 2018, 222 patients had been enrolled in a prospective observational study (study 2) to assess the association between PACU delirium and patient-reported outcomes representing the non-midazolam cohort (Kainz et al. 2022).

### Setting and participants

**Fig. 1:**
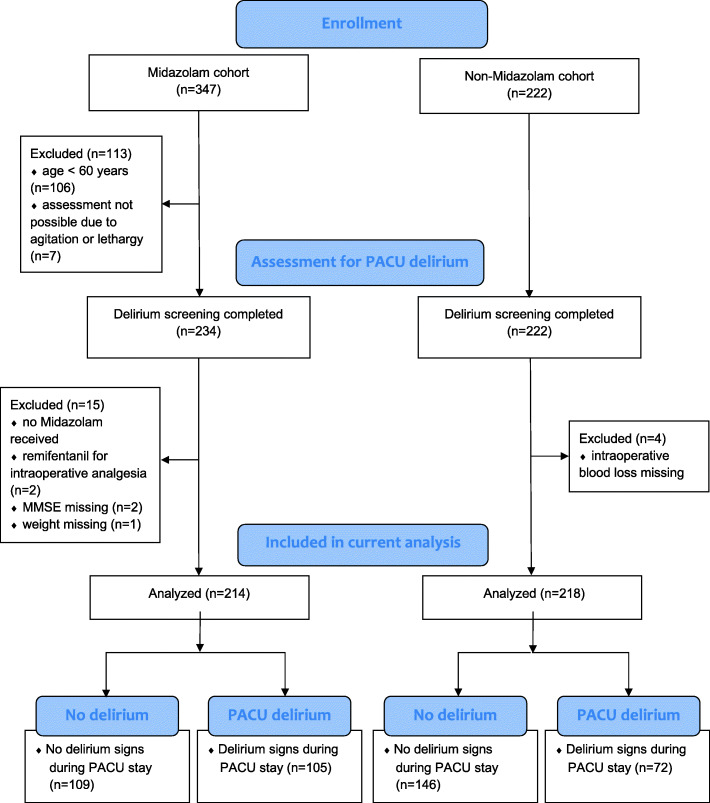
Flow of participants throughout the study. MMSE: mini-mental status examination. PACU: postanesthesia care unit

This study was performed at a high-volume prostate cancer center in Hamburg, Germany. Throughout the study period, approximately 2,500 radical prostatectomies were performed annually. The flow of participants is presented in Fig. 1. For the initial prospective cohort studies, patients were included, if they were scheduled for elective radical prostatectomy either by open retropubic or robot-assisted technique for treatment of prostate cancer and if they were fluent in German in order to undergo psychometric assessments (Beck et al. 2020a; Kainz et al. 2022). Study 1 included adult patients of any age; for study 2 we included patients of 60 years or older. Exclusion criteria were preexisting neurological disorders including cognitive impairment and dementia, or a history of cerebrovascular disease. Patients that were prescheduled for postoperative admission to the intensive care unit were excluded from study participation, since all postoperative assessments were performed during the PACU stay.

Participants from the two prospective cohort studies 1 and 2 were included in the current analysis, if they were 60 years or older and had completed the assessment for PACU delirium. Patients with contraindications to sedative premedication with midazolam such as obstructive sleep apnea and patients with preoperative anxiety were excluded.

### Premedication with midazolam

The midazolam cohort routinely received 7.5 mg oral midazolam for sedative premedication in the absence of contraindications. Premedication with midazolam was administered 30 to 60 min preoperatively. Since 2017, the liberal administration of midazolam prior to anesthesia and surgery has been restricted at our institution. Anesthetic premedication with midazolam has been administered in patients with preoperative anxiety and a score of 11 or more on the Amsterdam Preoperative Anxiety and Information Scale (Berth et al. 2007; Moerman et al. 1996).

### Anesthesiologic management

General anesthesia was induced with sufentanil (0.3–0.7 μg kg^−1^), propofol (2–3 mg kg^−1^), and rocuronium (0.6 mg kg^−1^). Anesthesia was maintained with sufentanil and sevoflurane (MAC target 0.8–1.2) or total intravenous anesthesia with sufentanil and propofol (4–7 mg kg^−1^ h^−1^). The choice of the anesthetic maintenance agent was at the discretion of the attending anesthesiologist. Anesthesia depth was monitored targeting a bispectral index (BIS™, Medtronic, MN) between 30 and 40. In the PACU, monitoring was continued (electrocardiogram, arterial blood pressure, peripheral oxygen saturation, urine output, arterial blood gas) and supplementary oxygen was administered as needed to maintain a peripheral oxygen saturation ≥ 94% (88–92% in patients with chronic obstructive pulmonary disease). If the numeric rating scale was 3 or higher, metamizole and/or piritramide were administered (Knipper et al. 2020).

### Assessment of delirium

The Confusion Assessment Method for the Intensive Care Unit (CAM-ICU) was used to determine the presence of PACU delirium. The CAM-ICU was performed 15, 30, 45, and 60 min after extubation by six members of the study team, who were trained in CAM-ICU assessment. The Richmond Agitation-Sedation Scale (RASS) was applied at the same time points to evaluate the severity of agitation or sedation. The RASS is a 10-point scale ranging from − 5 (unarousable) to + 4 (combative). The CAM-ICU was only applied in patients with a RASS score of − 3 or higher. We defined PACU delirium as a positive CAM-ICU at any one time point between 15 and 60 min after extubation.

### Data collection

On the day before surgery, patients were screened for depressive disorders with the Patient Health Questionnaire (PHQ-9). To screen for preexisting cognitive impairment, the Mini-mental Status Examination was administered preoperatively. Information on baseline demographic and clinical variables (age, body mass index, medical history, American Society of Anesthesiologists (ASA) physical status, and education), were obtained during baseline assessment on the day before surgery. Variables related to anesthesia and surgery were collected from the anesthesia protocol on the day of surgery. Patients with one or more missing variables were excluded from analysis (Fig. 1).

### Statistical analysis

Data are presented as mean ± standard deviation (SD) or absolute numbers and percentages, according to the measurement level of the data. Demographic and clinical characteristics as well as the incidence of PACU delirium in the historical and the new midazolam policy were compared with Mann-Whitney *U* tests, *χ*^2^ tests, or Fisher’s exact tests, as appropriate. CAM-ICU positivity at different time points was compared with *χ*^2^ tests or Fisher’s exact tests and Bonferroni corrected for multiple comparisons. We analyzed the association between preoperative medication with midazolam and PACU delirium using binary logistic regression. The multivariable model included ‘PACU delirium’ as the dependent variable and ‘midazolam cohort’ as the independent variable of primary interest. Other clinically relevant variables were included in the model as possible confounders: anesthetic agent for anesthesia maintenance (propofol vs. sevoflurane), type of surgery (open vs. robot-assisted radical prostatectomy), age, ASA physical status, estimated blood loss (ml), duration of surgery (min), sufentanil (μg kg^−1^ h^−1^), and the year of enrollment. With exception of ‘midazolam cohort’ and ‘year of enrollment’, which remained in the model, variables were eliminated stepwise backwards. ‘Duration of surgery’ and ‘estimated blood loss’ were transformed to their binary logarithm (*ln(x)/ln(2)*).

We performed a subgroup analysis to assess the association between premedication with midazolam and PACU delirium in patients who had received inhalational anesthesia with sevoflurane in combination with sufentanil. The multivariable model included the same variables as the main analysis except for the variable ‘type of anesthesia’ (propofol vs. sevoflurane).

SPSS Statistics 24 (IBM Deutschland GmbH) was used for statistical analyses. Figures were designed with GraphPad Prism 8 (GraphPad Software, San Diego, CA). This manuscript adheres to the STROBE reporting guidelines for observational studies.

## Results

### Patients’ characteristics and clinical variables

A total of 569 patients had been enrolled. Of these, 454 completed the assessment for PACU delirium and 432 (midazolam cohort: *n* = 214; non-midazolam cohort: *n* = 218) were included in the final analysis. Details on the flow of participants throughout the study are presented in Fig. 1. The mean age of the study population was 67 ± 4 years. The majority of patients (*n* = 373/432, 86.3%) fulfilled the criteria for ASA physical status I–II with significantly more ASA I and III patients in the midazolam cohort (*p* < 0.001). The duration of anesthesia (*p* < 0.001) and surgery (*p* = 0.002) and the estimated blood loss (*p* = 0.015) were significantly higher in the midazolam cohort compared with the non-midazolam cohort. Compared with participants from the midazolam cohort, patients in the non-midazolam cohort received sevoflurane for anesthesia maintenance more frequently (*p* < 0.001) and received significantly higher doses of sufentanil (*p* = 0.003). Scores from the Mini-mental status examination differed significantly between cohorts, without being clinically relevant. Details on baseline demographic and clinical characteristics of the study population are listed in Table 1.

**Table 1:** Patient charcteristics

	Midazolam cohort (*n* = 214)	Non-midazolam cohort (*n* = 218)	*P*
Year of enrollment			
2015	190 (88.8)		–
2016	24 (11.2)		–
2017		39 (17.9)	–
2018		179 (82.1)	–
Baseline characteristics			
Age, years	67 ± 4	67 ± 5	0.204
Body mass index	26.7 ± 3.5	26.2 ± 3.1	0.212
Education			0.064
Graduation from highschool	118 (55.1)	117 (53.7)	
No graduation from highschool	91 (42.5)	101 (46.3)	
Not available	5 (2.3)	0 (0.0)	
Mini-mental Status Examination	29 ± 1	29 ± 1	< 0.001
Patient Health Questionnaire 9	4 ± 3	3 ± 3	0.197
ASA physical status			< 0.001
I	43 (20.1)	21 (9.6)	
II	133 (62.1)	176 (80.7)	
III	38 (17.8)	21 (9.6)	
Comorbid conditions			
Arterial hypertension	113 (52.8)	122 (56.0)	0.510
Coronary heart disease	25 (11.7)	20 (9.2)	0.394
Congestive heart failure	2 (0.9)	3 (1.4)	1.000
Chronic kidney disease	7 (3.3)	2 (0.9)	0.104
Diabetes mellitus	11 (5.1)	17 (7.8)	0.262
Dyslipoproteinemia	59 (27.6)	56 (25.7)	0.700
Chronic obstructive pulmonary disease	10 (4.7)	5 (2.3)	0.199
Current smoking status	17 (7.9)	17 (7.8)	0.955
Surgery and anesthesia			
Type of surgery			0.562
Robot-assisted radical prostatectomy	103 (48)	111 (51)	
Open retropubic radical prostatectomy	111 (52)	107 (49)	
Duration of surgery, min	191 ± 46	177 ± 33	0.002
Estimated blood loss, ml	670 ± 522	542 ± 404	0.015
Anesthesia maintenance			< 0.001
Sevoflurane	121 (57)	216 (99)	
Propofol	93 (44)	2 (1)	
Duration of anesthesia, min	272 ± 53	250 ± 40	< 0.001
Administered fluids, ml	2738 ± 816	2573 ± 636	0.054
Sufentanil (μg kg^−1^ h^−1^)	0.23 ± 0.05	0.25 ± 0.05	0.003

Baseline characteristics and variables related to anesthesia and surgery in patients, who received midazolam preoperatively (midazolam cohort) and patients without midazolam for sedative premedication (non-midazolam cohort)

### Incidence of postanesthesia care unit delirium

**Fig. 2:**
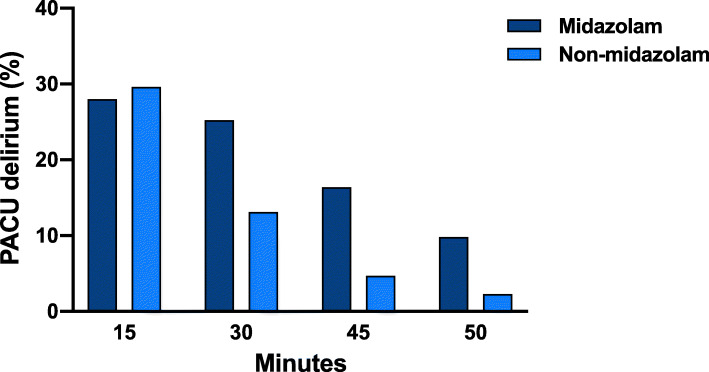
Positive screening for postanesthesia care unit (PACU) delirium 15, 30, 45, and 60 min after extubation, stratified for premedication with and without midazolam

Of 214 patients, who had received midazolam, 105 (49.1%) tested positive for delirium at any one time point during the PACU stay. In the non-midazolam cohort 72/218 patients (33.0%) showed delirium signs in the PACU (Table 2). The incidence of PACU delirium differed significantly between the historical and the new midazolam policy (*P* < 0.001, Supplementary Figure 1). The incidence of PACU delirium was highest at 15 min following extubation and decreased during the PACU stay in both groups (Fig. 2). At 30 min, 45 min, and 60 min significantly more patients in the midazolam cohort were screened positive for PACU delirium compared with patients, who had not received midazolam for premedication (Fig. 2).

**Table 2:** Assessment of postanesthesia care unit delirium

	Midazolam cohort (*n* = 214)	Non-midazolam cohort (*n* = 218)
PACU delirium	105 (49.1)	72 (33.0)
Positive CAM-ICU assessments		
1	57 (26.6)	43 (19.7)
2	38 (17.8)	25 (11.5)
3	8 (3.7)	3 (1.4)
4	2 (0.9)	1 (0.5)

Postanesthesia care unit (PACU) delirium was defined as a positive Confusion Asessment Method for the Intensive Care Unit (CAM-ICU) at any one time point during the PACU stay. The numer of positive assessments is presented for each cohort. Data are given as absolute and relative numbers

### Association between premedication with midazolam and postanesthesia care unit delirium

Multivariable analysis did not show a significant association between premedication with midazolam and PACU delirium (OR 0.847 [95% CI 0.164; 4.367]; *P* = 0.842). Age (OR 1.102 [95% CI 1.050; 1.156]; *P* < 0.001), the cumulative dose of sufentanil (OR 1.014 [95% CI 1.005; 1.024]; *P* = 0.005), and total intravenous anesthesia (TIVA) for anesthesia maintenance (OR 2.805 [95% CI 1.497; 5.256]; *P* = 0.001) were significantly associated with PACU delirium (Table 3).

**Table 3:** Binary logistic regression analysis

	OR	95% CI	*P*
First step			
Midazolam (reference: no midazolam)	0.720	0.128; 4.067	0.710
Year of enrollment (per year increase)	0.805	0.429; 1.512	0.500
Propofol (reference: sevoflurane)	2.653	1.395; 5.044	0.003
ORP (reference: RARP)	1.061	0.583; 1.934	0.846
Age (per year increase)	1.098	1.046; 1.152	< 0.001
ASA physical status (reference: ASA III)			
ASA I	0.907	0.499; 1.649	0.749
ASA II	1.286	0.595; 2.778	0.523
Estimated blood loss (per ml increase)	1.165	0.828; 1.638	0.381
Duration of surgery (per min increase)	2.028	0.657; 6.260	0.219
Sufentanil (per μg kg^−1^ h^−1^ increase)	1.014	1.002; 1.025	0.018
Final step			
Midazolam (reference: no midazolam)	0.847	0.164; 4.367	0.842
Year of enrollment (per year increase)	0.829	0.458; 1.499	0.535
Propofol (reference: sevoflurane)	2.805	1.497; 5.256	0.001
Age (per year increase)	1.102	1.050; 1.156	< 0.001
Sufentanil (per μg kg^−1^ h^−1^ increase)	1.014	1.005; 1.024	0.004

Factors associated with postanesthesia care unit delirium were analyzed with binary logistic regression. Variables were eliminated stepwise backwards with the first and the final step presented. ‘Midazolam’ and ‘year of enrollment’ were forced into the model. ‘Duration of surgery’ and ‘estimated blood loss’ were transformed to their binary logarithm (*ln(x)/ln(2)*). *RARP* robot-assisted radical prostatectomy. *ORP* open retropubic radical prostatectomy

### Subgroup analysis

Of 337 patients with sevoflurane-sufentanil for anesthesia maintenance, 121 patients had received midazolam and 216 patients had not. In the midazolam cohort, 49/337 (40.5%) patients tested positive for PACU delirium. A total of 71/337 (32.9%) patients in the non-midazolam cohort showed signs of PACU delirium. Multivariable analysis did not reveal a significant association between midazolam and PACU delirium (OR 1.240 [95% CI 0.241; 6.386]; *P* = 0.797). Age (OR 1.100 [95%CI 1.043; 1.161]; *P <* 0.001) and duration of surgery (OR 4.123 [95% CI 1.284; 13.241]; *P* = 0.017) were associated with the development of PACU delirium (Table 4).

**Table 4:** Subgroup analysis

	OR	95% CI	P
First step			
Midazolam (reference: no midazolam)	0.805	0.140; 4.620	0.808
Year of enrollment (per year increase)	0.823	0.438; 1.566	0.563
ORP (reference: RARP)	1.163	0.595; 2.169	0.700
Age (per year increase)	1.103	1.044; 1.164	< 0.001
ASA physical status (reference: ASA III)			
ASA I	0.872	0.435; 1.748	0.699
ASA II	1.140	0.468; 2.776	0.772
Estimated blood loss (per ml increase)	1.093	0.752; 1.588	0.642
Duration of surgery (per min increase)	2.661	0.668; 10.597	0.165
Sufentanil (per μg kg^−1^ h^−1^ increase)	1.010	0.996; 1.024	0.145
Final step			
Midazolam (reference: no midazolam)	1.240	0.241; 6.386	0.797
Year of enrollment (per year increase)	0.933	0.513; 1.695	0.819
Age (per year increase)	1.100	1.043; 1.161	< 0.001
Duration of surgery (per min increase)	4.123	1.284; 13.241	0.017

Subgroup analysis of 337 patients who received sevoflurane for anesthesia maintenance with postanesthesia care unit delirium as the dependent variable. ‘Midazolam’ and ‘year of enrollment’ were forced into the model. ‘Duration of surgery’ and ‘estimated blood loss’ were transformed to their binary logarithm (*ln(x)/ln(2)*). *RARP* robot-assisted radical prostatectomy. *ORP* open retropubic radical prostatectomy

## Discussion

The aim of this study was to compare the incidence of PACU delirium between patients who had received oral midazolam preoperatively and patients who had not been administered any sedative medication prior to anesthesia induction. Compared with the midazolam cohort, the incidence of PACU delirium was significantly lower in patients under the new policy without sedative premedication. Contrary to our hypothesis, we did not find a significant association between premedication with midazolam and PACU delirium in multivariable analysis. Age, sufentanil, and TIVA for anesthesia maintenance were significantly associated with PACU delirium.

Prevention of delirium removes a tremendous burden from patients, their families, and caregivers and may help to substantially reduce morbidity, mortality, and health care cost. It is thus of utmost importance to identify and eliminate factors that trigger delirium. There is a strong association between benzodiazepines, administered postoperatively or prolonged benzodiazepine use, and delirium status that has been demonstrated in numerous studies (Fraser et al. 2013; Maldonado et al. 2009; Pandharipande et al. 2006; Zaal et al. 2015). However, the role of premedication with benzodiazepines in the development of PACU delirium is not well understood. Importantly, the single use of benzodiazepines to reduce preoperative anxiety has not been linked with POD so far (Wang et al. 2021). In a prospective study, Radtke et al. observed that premedication with benzodiazepines was a risk factor for inadequate emergence after anesthesia (Radtke et al. 2010). Similarly, data from an observational study showed an association between benzodiazepines and agitation during the immediate postoperative period (Lepousé et al. 2006). By contrast, Card and colleagues did not observe a statistical association between midazolam equivalents and delirium features during the PACU stay (Card et al. 2015). In line with these results, we did not find a significant association between premedication with midazolam and PACU delirium in multivariable analysis. It is plausible that preoperative benzodiazepines may contribute to inadequate emergence and prolonged recovery after anesthesia without necessarily causing subsequent POD (Lepousé et al. 2006; Radtke et al. 2010). A randomized controlled trial that compares liberal and restrictive premedication with midazolam with respect to PACU delirium is urgently needed.

We found a significant association between TIVA for anesthesia maintenance and a higher incidence of PACU delirium. Data on the influence of anesthetic substances on perioperative neurocognitive disorders is contradictory. Single studies report an association between inhalational anesthetics and PACU delirium (Ishii et al. 2016; Munk et al. 2016). In a recent meta-analysis, the incidence of POD was compared between propofol-based TIVA and inhalational agents for anesthesia maintenance. Interestingly, the authors did not find a beneficial effect of either substance on the development of POD (Miller et al. 2018). In line with our results, several studies report on the association between propofol for anesthesia maintenance and perioperative neurocognitive disorders (Hesse et al. 2019; Schoen et al. 2011). Interestingly, data from an in vitro study suggest strong anticholinergic properties including epigenetic modifications under the influence of propofol that may provide an explanation for the positive association between propofol use and delirium (Holtkamp et al. 2019). It is important to note that our study was not primarily designed to detect a difference in delirium signs between two different anesthetic regimens. Therefore, our findings on the association between TIVA and PACU delirium should be interpreted with caution.

We performed a subgroup analysis to assess whether a potential association between midazolam and PACU delirium may have been confounded by the strong association between propofol and PACU delirium. However, we did not find a significant association between midazolam and signs of delirium in patients, who had received inhalational anesthesia.

### Strengths and limitations

Studies on the incidence of PACU delirium vary substantially with regard to screening instruments used and time points of assessment (Card et al. 2015; Hernandez et al. 2017; Hesse et al. 2019; Saller et al. 2020). Methodological heterogeneity among trials on PACU delirium may limit the comparability of study results. One strength of this study is the repetitive assessment at four predefined time points during the PACU stay (15, 30, 45, and 60 min postoperatively). Moreover, all patients were screened by one team of six investigators. However, delirium screening in our study was limited to the postopeorative recovery period in the PACU and we cannot generalize our results to POD beyond the PACU stay. We used the CAM-ICU, which is a validated and widely used tool for the assessment of POD (Card et al. 2015; Hesse et al. 2019; Saller et al. 2020). Despite high specificity, sensitivity for delirium signs in the PACU may be limited (Neufeld et al. 2013b). Therefore, we may have even underestimated the incidence of PACU delirium. Screening instruments that are more suitable for the PACU environment have been developed after patients had been enrolled in this study (Hight et al. 2018; Olbert et al. 2019). Future studies on PACU delirium should use novel screening instruments that take into account the specific circumstances of the PACU setting.

This secondary analysis of prospectively collected data before and after implementation of a restrictive benzodiazepine policy was neither randomized nor blinded. Thus, the nature of the study design only allows for exploratory analysis. Participants were enrolled between 2015 and 2018. To reduce history bias, we included the year of patient enrollment as a potential confounding factor on midazolam and PACU delirium in the statistical analysis. It is important to note, however, that this might not sufficiently reflect all advances of perioperative management throughout the study period.

The lack of association between midazolam and PACU delirium might be attributable to the relatively low perioperative risk of our study population, the majority being categorized as ASA physical status I or II. Moreover, patients with a history of cerebrovascular or neurodegenerative disease including mild cognitive impairment or dementia had been excluded from study participation. Therefore, we may have missed the patients most susceptible to neurocognitive adverse effects of benzodiazepines. It is possible, that the role of benzodiazepines as a risk factor for delirium becomes more relevant in patients with pre-existing neurocognitive impairment who have a high baseline vulnerability (Marcantonio et al. 1994). We only included patients undergoing prostatectomy. While the homogeneity of the baseline characteristics among cohorts is certainly one strength of our study, generalizability of the results may be limited.

We administered oral midazolam in the midazolam cohort. Oral midazolam is absorbed reliably and reaches peak plasma concentrations after around 60 min (Smith et al. 1981). Its major disadvantage is reduced bioavailability due to the hepatic first-pass effect. However, the prolonged time to reach peak plasma concentrations and to exert clinical effects allows for application before transfer to the surgical area. Despite a difference in absorption and reduced bio-availability of oral midazolam between 35 and 44%, clearance and terminal half-life values do not differ substantially between intravenous and oral midazolam application (Smith et al. 1981). Therefore, the route of midazolam administration should not affect the occurrence of delirium.

## Conclusion

Over a 4-year period we observed a significant decrease in PACU delirium from 49% to 33% in elderly patients, who underwent radical prostatectomy. Of note, midazolam for sedative premedication was not significantly associated with PACU delirium. The reduction in the incidence of PACU delirium throughout the study period between 2015 and 2018 may be attributable to unobserved variables, such as improvements in perioperative management, other than a more restrictive preoperative benzodiazepine administration.

## Supplementary Information

**Additional file 1: Supplementary Figure 1.** Postanesthesia care unit (PACU) delirium in patients who received midazolam for sedative premedication (midazolam cohort) and patients without preoperative midazolam administration (non-midazolam cohort).

## Data Availability

The datasets used and analyzed during the current study are available from the corresponding author on reasonable request.

## Abbreviations

ASA: American Society of Anesthesiologists
BIS: Bispectral index (BIS™)
CAM-ICU: Confusion Assessment Method for the Intensive Care Unit
PHQ-9: Patient Health Questionnaire
PACU: Postanesthesia care unit
POD: Postoperative delirium
RASS: Richmond Agitation-Sedation Scale
TIVA: Total intravenous anesthesia
