# Supplementary figures and images for: Effect of sedative premedication with oral midazolam on postanesthesia care unit delirium in older adults: a secondary analysis following an uncontrolled before-after design
Source: Perioper Med (Lond). 2022 May 19;11:18. doi: 10.1186/s13741-022-00253-4 (PMC9118741; doi:10.1186/s13741-022-00253-4)

# PACU delirium before and after restriction of midazolam for sedative premedication

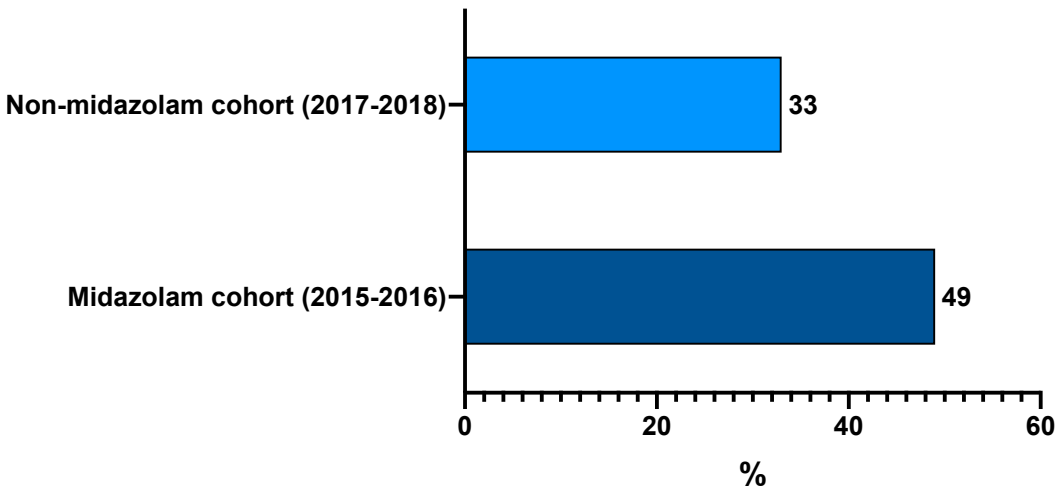

Supplement: Supplementary file 1 — Additional file 1: Supplementary Figure 1. Postanesthesia care unit (PACU) delirium in patients who received midazolam for sedative premedication (midazolam cohort) and patients without preoperative midazolam administration (non-midazolam cohort). [file 13741_2022_253_MOESM1_ESM.pdf]
